# Spatio-Temporal Evolution Analysis of Drought Based on Cloud Transformation Algorithm over Northern Anhui Province

**DOI:** 10.3390/e22010106

**Published:** 2020-01-16

**Authors:** Xia Bai, Yimin Wang, Juliang Jin, Shaowei Ning, Yanfang Wang, Chengguo Wu

**Affiliations:** 1 State Key Laboratory of Eco-Hydraulics in Northwest Arid Region, Xi’an University of Technology, Xi’an 710048, China; baixia0516@163.com; 2 School of Civil and Hydraulic Engineering, Bengbu University, Bengbu 233030, China; wule9825@163.com; 3 School of Civil Engineering, Hefei University of Technology, Hefei 230009, China; jinjl66@126.com (J.J.); ning@hfut.edu.cn (S.N.); wucguo@outlook.com (C.W.)

**Keywords:** drought evolution analysis, uncertainty, entropy, cloud transformation algorithm, conceptual zooming, Northern Anhui province

## Abstract

Drought is one of the most typical and serious natural disasters, which occurs frequently in most of mainland China, and it is crucial to explore the evolution characteristics of drought for developing effective schemes and strategies of drought disaster risk management. Based on the application of Cloud theory in the drought evolution research field, the cloud transformation algorithm, and the conception zooming coupling model was proposed to re-fit the distribution pattern of SPI instead of the Pearson-III distribution. Then the spatio-temporal evolution features of drought were further summarized utilizing the cloud characteristics, average, entropy, and hyper-entropy. Lastly, the application results in Northern Anhui province revealed that the drought condition was the most serious during the period from 1957 to 1970 with the SPI12 index in 49 months being less than −0.5 and 12 months with an extreme drought level. The overall drought intensity varied with the highest certainty level but lowest stability level in winter, but this was opposite in the summer. Moreover, drought hazard would be more significantly intensified along the elevation of latitude in Northern Anhui province. The overall drought hazard in Suzhou and Huaibei were the most serious, which is followed by Bozhou, Bengbu, and Fuyang. Drought intensity in Huainan was the lightest. The exploration results of drought evolution analysis were reasonable and reliable, which would supply an effective decision-making basis for establishing drought risk management strategies.

## 1. Introduction

Drought is an extreme climate event. The conceptual definition of drought mainly focuses on the essential characteristics of a drought process while the descriptive definition of drought is primarily linked with the occurrence, recognition, and evolution of the drought event [1]. Overall, drought can be defined as a random environmental disaster and is mainly characterized with a water deficit, which has a critical and far-reaching impact on agriculture, social economy, and ecosystem, and has attracted more attention from meteorologists and hydrologists [1,2]. In recent years, with the influence of global climate change, extreme climate events occur more frequently, and the frequency and intensity of drought also present an evident increasing trend [1,2,3]. Spatio-temporal evolution characteristics of drought is a primary factor that leads to the disequilibrium distribution of regional water resources availability, which will, conversely, have a great impact on regional water resources development, utilization, and an ecological environment [2,4,5,6]. Therefore, the understanding of spatio-temporal distribution characteristics of drought is of significant importance for exploring the evolution features of drought and then establishing feasible and effective drought disaster regulation strategies. To date, the most widely-applied research method of drought evolution trend mainly focuses on the stochastic process and statistical related theory. Drought evolution characteristics is usually described by various indicators [4,6,7,8,9], and plenty of research and investigation involving the causing factors, distribution, and evolution features of drought from the perspective of hydrological, meteorological, and remote sensing have been carried out around the world. For instance, T. B. McKee et al. (1993) proposed a Standard Precipitation Index (SPI) to describe the variation characteristics of drought and flood disasters from different time scales [10]. Y. Zhang et al. (2008) discussed drought and flood variation characteristics during the pre-flood season by the mathematical statistics method based on the daily precipitation data from 1958 to 2004 in Huanan Station [11]. W. H. Huang et al. (2010) analyzed the ratio of the station number with drought to the total station number from annual and seasonal scales based on a calculated SPI value, which revealed that the impact of drought on agricultural production would be more serious in Southern China [12]. Y. L. Ren et al. (2013) analyzed drought evolution characteristics based on SPI from a monthly scale using the monthly precipitation data from 1961 to 2009 from 137 hydrological stations in Northwest China [13].

Based on the above, it can be seen that an existing drought indicator can be effectively applied to describe drought evolution features from a specific perspective. However, how to construct a comprehensive drought indicator to capture the hydrologic and meteorological uncertainties is still a challenge for drought risk assessment research [14,15]. Moreover, the key of exploration of drought variation characteristics can be focused on the recognition and quantification of uncertainties throughout the drought evolution process by an integrated drought indicator. Cloud model related theory, as a reliable tool of mathematical transformation from conceptual qualitative description to its quantitative computation, has been widely applied in many fields, including image recognition, intelligent modeling, big data analysis, and more [16,17]. Concerning complicated uncertainties throughout the drought variation process and the systematic analysis approaches themselves, the primary motivation of this manuscript is to explore the spatio-temporal evolution characteristics of drought by transforming the calculated SPI series data into qualitative concepts of Gaussian cloud distribution, and then realizing the soft division of drought grade conceptual clouds through the Cloud Transformation Algorithm (CTA) and the Conception Zooming method. Ultimately, the proposed approach was applied in the case study analysis in the Anhui province of China to testify its reliability and effectiveness, and can be further extended to provide a new research idea of the drought process analysis.

## 2. Introduction of Drought in Northern Anhui Province

**Figure 1:**
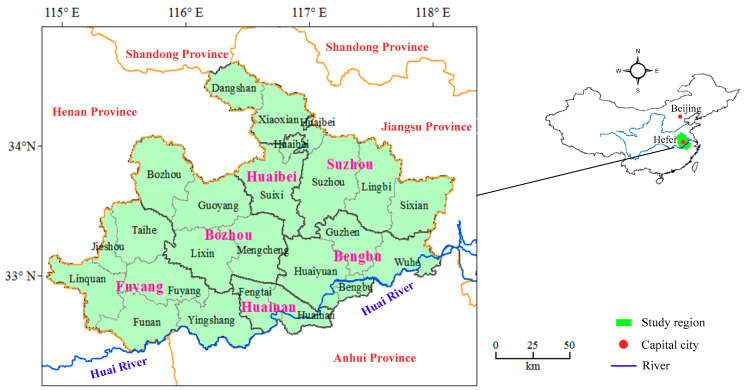
Geographical location of the Northern Anhui province in China.

As indicated in Figure 1, the Northern Anhui province, located in the hinterland of East China and North of Huai River with longitude between 114°58′ and 18°10′ and latitude between 32°45′ and 34°35′, has jurisdiction over six cities including Suzhou, Bozhou and Bengbu and 17 counties including Dangshan, Lingbi, and Huaiyuan. The Northern Anhui province is mainly plain with a total area of 36,694 km^2^, a natural slope between 0.13% and 0.83%, and an average altitude between 20 m and 40 m. In addition, the Northern Anhui province is located in the transition zone between the north subtropical humid climate and half of the moist monsoon climate. The annual average precipitation is between 770 mm to 950 mm, but varies significantly both from spatial and temporal scales, which presents an increasing trend from north to south. Moreover, the annual average precipitation during the flood season from June to September accounts for 70% to the total mainly with the form of rainstorm, which is beneficial for the occurrence of waterlogging in the flood season and drought in the non-flood season. Water shortage is also serious in the North Anhui province with the annual average water resources availability of 12.87 billion m^3^, which is half of the provincial level and even below 0.75 of the average national level. Compared with the surface water resources availability, the ground water resources are abundant, and the ratio for surface and ground water resources is approaching 1: 1 in the North Anhui province.

For the dual influences of precipitation and geomorphic factors, the Northern Anhui province is one of the most vulnerable areas suffering from drought-related disasters, according to the historical statistics and national standard of meteorological drought classification criteria [5,6]. A total of 109 droughts occurred during 500 years from 1450 to 1949 with an occurring frequency of about 4 years, and another 36 autumn droughts occurred during 64 years after the foundation of PRC, in which 5 years had extreme droughts and 14 years had extraordinary droughts. Furthermore, drought duration in Northern Anhui province is long, and frequently follows with waterlogging. Thus, it is evident that drought is the most common and heavily damaged natural disasters in the Northern Anhui province, and drought evolution characteristic analysis is urgent and fundamental for carrying out risk comprehensive management of drought disaster.

## 3. Methodologies

### 3.1. Standardized Precipitation Index (SPI)

The drought indicator is an effective tool to explore the variation characteristics of the drought evolution process, which is also a crucial tool to measure the influence of drought to a regional socio-economic system [18]. The Standardized Precipitation Index (SPI) is a typical meteorological drought indicator, which has an ability to reveal statistical distribution rules of precipitation by meteorological theory, and then derive the magnitude, intensity, and duration of the drought process [18,19]. The SPI was initially proposed and applied to evaluate the drought evolution in Colorado State by Mckee in 2002 [10,20], and now is accepted by the World Meteorological Organization as the reference drought index for effective drought monitoring and evaluation [21]. The SPI computation is done by fitting historical precipitation data to a Gamma probability distribution function for a specific time period and location. The detailed calculation procedures of SPI can be described as follows [21,22].

Step 1: Suppose the precipitation data series of a specific time period satisfies Gamma distribution. Then its probability distribution function can be denoted as Equation (1).

(1) g(x)=1βαΓ(α)xα−1e−xβ

where *α* and *β* are the shape and scale parameters, respectively, which satisfy *α* > 0 and *β* > 0, and usually can be estimated using the maximum likelihood method, as follows.

(2) $$\alpha =\frac{1}{4A}(1+\sqrt{1+4A/3})$$

(3) $$\beta =\bar{x}/\alpha$$

(4) $$A=\ln(\bar{x})-\frac{\sum_{i=1}^{n}\ln(x_{i})}{n}$$

where parameter *n* and $\bar{x}$ are the length and average of precipitation data series, respectively. Based on the parameter calibration, the cumulative possibility of precipitation *x* not exceeding a specific value *x*_0_ in a given time scale can be determined as follows.

(5) G(x<x0)=∫0xg(x)dx=∫0x1βα^Γ(α^)xα^−1e−xβ^ dx

Step 2: the definition in Equation (5) is not valid when precipitation *x* equals 0. Thus, the cumulative possibility *G*(*x* < *x*_0_) should be modified as follows.

(6) H(x)=q+(1−q)⋅G(x)

where *q* is the possibility when precipitation *x* equals 0, which can be obtained by *q* = *m*/*n*, and parameter *m* is the sample number with precipitation *x* equal to 0. 

Step 3: Normal standardization processing. Through numerical integration computation, the calculated cumulative probability *H*(*x*) can be transformed to a standard normal distribution to yield SPI, which can be denoted as follows [22,23].

(7) $$SPI=\left\{ \begin{array}{l} \frac{c_{0}+c_{1}\cdot t+c_{2}\cdot t^{2}}{1+d_{1}\cdot t+d_{2}\cdot t^{2}+d_{3}\cdot t^{3}}\;(0<H(x)\leq 0.5)\\ t-\frac{c_{0}+c_{1}\cdot t+c_{2}\cdot t^{2}}{1+d_{1}\cdot t+d_{2}\cdot t^{2}+d_{3}\cdot t^{3}}\;(0.5<H(x)\leq 1) \end{array}\right.$$

(8) $$t=\left\{ \begin{array}{l} \sqrt{\ln\frac{1}{H{\left(x\right)}^{2}}}\;(0<H(x)\leq 0.5)\\ \sqrt{\ln\frac{1}{{\left[1-H(x)\right]}^{2}}}\;(0.5<H(x)\leq 1) \end{array}\right.$$

where *c*_0_ = 2.515517, *c*_1_ = 0.802853, *c*_2_ = 0.010328, *d*_1_ = 1.432788, *d*_2_ = 0.189269, and *d*_3_ = 0.001308.

### 3.2. Forward Normal Cloud Algorithm (FNCA)

Proposed by Chinese scholar D. Y. Li, the Cloud model is an effective mathematical cognitive tool to describe the transforming mechanism of uncertainty from a qualitative concept to its corresponding quantitative expression utilizing three parameters including average, entropy, and hyper-entropy [16]. The mathematical definition of cloud drop and its certainty degree can be explained as follows [16,24]. 

**Figure 2:**
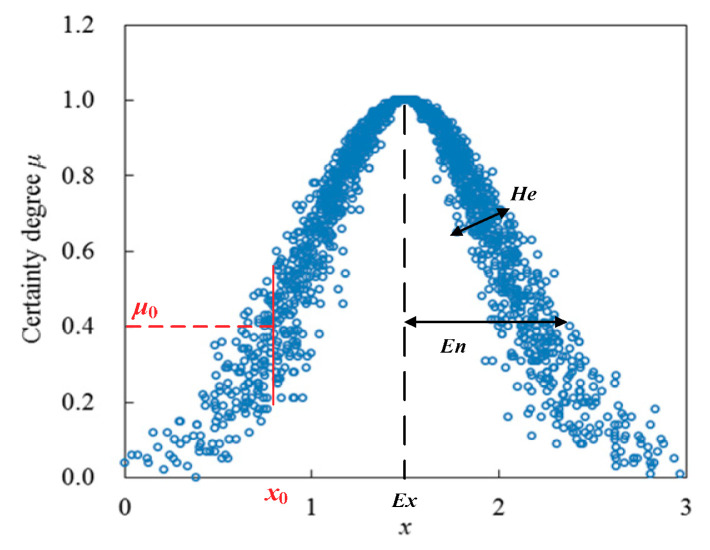
Distribution of a qualitative concept cloud *C*(1.5, 0.5, 0.1).

Suppose *U* is a universal set denoted by precise data, and *C* is the qualitative concept related to *U*. If randomly generated data *x* (*x* ∈ *U*) is related to concept *C*, then the membership degree of random data *x* belonging to concept *C* is named as a certainty degree [16,24]. Specifically, let *C*(*Ex*, *En*, *He*) be a qualitative concept *C* related to precise set *U* and described by average *Ex*, entropy *En*, and hyper-entropy *He* in which average *Ex* denotes the most representative sample data of concept *C*, and can be adopted to reveal the concentration condition of samples. Entropy *En* is related to the uncertainty of sample distribution and reflects both the random dispersing degree of cloud drops and acceptable range of concept *C*. Hyper-entropy *He* is the uncertainty degree of entropy, which reflects the concentration condition of samples under a certain deviation condition [16]. Thereby, if the distribution of variable *x* satisfies *x* ∈ *N*(*Ex*, *En*′^2^) and *En***^′^** ∈ *N*(*En*, *He*^2^), then the distribution of variable *x* within set *U* is defined as one-dimensional normal cloud, and data point [*x*_0_, *μ*_0_(*x*)] is defined as the cloud drop [16,24]. Figure 2 illustrates the distribution of cloud *C*, which satisfies *Ex* = 1.5, *En* = 0.5, and *He* = 0.1.

In practice, the most widely applied form of Cloud theory are forward and backward normal cloud algorithms. The forward normal cloud algorithm (FNCA) explores the sample distribution basing on determined cloud characteristic parameters *Ex*, *En*, and *He*, while the backward normal cloud algorithm (BNCA) is opposite, which is primarily applied to estimate the cloud characteristic parameters *Ex*, *En*, and *He* in terms of a statistical perspective [16,25]. The procedure of one-dimensional FNCA can be described as follows.

Step 1: randomly generate normally distributed variable *En*′, which satisfies *En*′~*N*(*En*, *He*^2^).

Step 2: randomly generate normally distributed variable *x_i_*, which satisfies *x*~*N*(*Ex*, *En*′^2^).

Step 3: calculate the certainty degree *μ* of data *x* belonging to the concept *C* through Equation (9).

(9) $$\mu =\;\exp\left[-\frac{{\left(x-Ex\right)}^{2}}{2{\left(En\prime \right)}^{2}}\right]$$

Step 4: repeat step 1 to step 3 until generating *N* cloud drops, and then the cloud distribution of concept *C* can be obtained.

### 3.3. Cloud Transformation Algorithm (CTA) and Conception Zooming

The precondition for the application of BNCA and FNCA assumes all given samples belong to one specific qualitative concept, which will restrict the exploration of more possible properties and knowledge related to the sample distribution [16]. It has been testified that the curve fitting error can be reduced clearly by dividing the overall probability density function into the combination of several normal distributions [16,17]. The cloud transformation algorithm (CTA), as an effective tool of probability statistical analysis, is capable of transforming overall probability density function into the integration of multiple Gaussian cloud distribution, which can be applied for the generation and division of a complicated concept [16,17,26]. CTA can be interpreted as the exploration of concept attributes from its actual sample distribution in a more refined scale [26]. In other words, the data sample with higher frequency will also have a higher contribution to the qualitative concept. Therefore, the local maximum of data frequency distribution can be regarded as the center of a qualitative concept (expectation value of concept cloud). Then, we substract the corresponding sample in the original sample distribution and calculate the next local maximum. Lastly, we repeat the above procedures until the frequency of the remained data sample is below a specific threshold [17,26]. Considering the drought evolution process can also be described through SPI, the CTA method based on peak theory was introduced in the drought spatio-temporal evolution research field in this study [16,17].

One of the key issues when applying the CTA method for curve fitting is the determination of entropy values *En_i_*. After the determination of average *Ex_i_*, the difference frequency value *δ* between *Ex_i_* and the remaining probability density peak can be obtained, if the difference value *δ* is larger than the specific threshold *μ*. Then average data *Ex_i_*′ with the shortest distance to *Ex_i_* can be obtained, and the corresponding entropy *En_i_* and hyper-entropy *He_i_* can be determined according to the sample distribution within the interval [*Ex_i_*-fabs(*Ex_i_*′-*Ex_i_*), *Ex_i_*-fabs(*Ex_i_*′-*Ex_i_*)] through the BNCA method [27,28]. In addition, to better fit the probability distribution curve of SPI, the entropy *En_i_* corresponding to a different conceptual cloud also need to be adjusted slightly [28].

Generally, because of not considering the relationship between different cloud distribution patterns, the initial conceptual cloud distribution derived from the CTA method is rough, which might lead to the presentence of a closely distributed cloud pattern or “blank area” [16,17,26]. Thereby, the initial conceptual cloud distribution derived from the CTA method need to be further improved by the zooming process. Conceptual cloud zooming is frequently accomplished by merging the neighboring cloud distribution patterns to simplify the cloud transformation results [16]. Considering the influence of the amplitude coefficient for different conceptual cloud distribution, the two neighboring cloud distribution patterns can be merged based on their intersecting level [29,30]. Suppose the two neighboring cloud distributions can be denoted as *C*_1_(*Ex*_1_, *En*_1_, *He*_1_) and *C*_2_(*Ex*_2_, *En*_2_, *He*_2_), and their expectation curves intersect at point *A*(*x_d_*, *y_d_*). Then the truncation entropy *En*_1_**^′^** and *En*_2_′ can be determined as follows [17,29,30].

(10) $$En_{1}^{\prime }=\frac{1}{\sqrt{2\pi }}\int_{-\infty }^{x_{d}}r_{1}e^{-{\left(x-Ex_{1}\right)}^{2}/\left(2En_{1}{}^{2}\right)}dx$$

(11) $$En_{2}^{\prime }=\frac{1}{\sqrt{2\pi }}\int_{xd}^{+\infty }r_{2}e^{-{\left(x-Ex_{2}\right)}^{2}/\left(2En_{2}{}^{2}\right)}dx$$

where *r*_1_ and *r*_2_ are the amplitude coefficients of cloud distribution *C*_1_ and *C*_2_, respectively, and the final characteristic values of emerged cloud distribution can be obtained as follows [17,29].

(12) $$Ex_{3}=\frac{Ex_{1}\cdot En_{1}^{\prime }+Ex_{2}\cdot En_{2}^{\prime }}{En_{1}^{\prime }+En_{2}^{\prime }}$$

(13) $$En_{3}=\frac{En_{1}^{\prime }}{r_{1}}+\frac{En_{2}^{\prime }}{r_{2}}$$

(14) $$He_{3}=\frac{He_{1}\cdot En_{1}^{\prime }+He_{2}\cdot En_{2}^{\prime }}{En_{1}^{\prime }+En_{2}^{\prime }}$$

Furthermore, the amplitude coefficients *r*_3_ of emerged cloud distribution can be obtained as follows.

(15) $$r_{3}=\frac{En_{1}{}^{\prime }+En_{2}{}^{\prime }}{En_{3}}$$

### 3.4. Analysis Framework of this Manuscript

**Figure 3:**
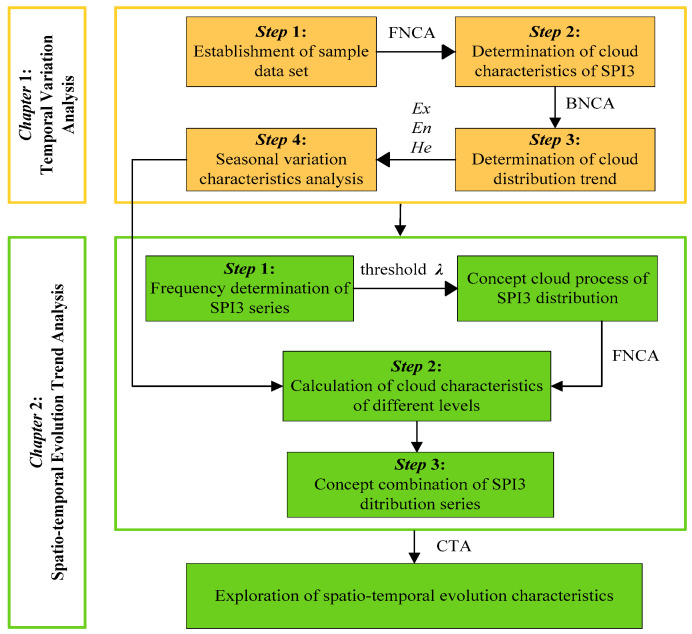
Framework of drought evolution characteristic analysis of this study.

The primary innovation of this study is to explore the spatial-temporal distribution features of drought in the Northern Anhui province from seasonal scale through re-fitting the distribution pattern of the SPI index via a cloud transformation algorithm instead of Pearson-III distribution. Thus, in the first section, the approaches applied in this paper were briefly introduced. In the second section, based on the frequency analysis of historical SPI series, the initial cloud characteristic parameters were determined. Then, concerning the similarity of different cloud patterns, the ultimate cloud distribution modes were obtained by conception zooming and merging, which were applied to reveal the spatial-temporal distribution features of drought. Thus, the analyzing framework of this manuscript can be illustrated in Figure 3.

## 4. Results and Discussion

**Figure 4:**
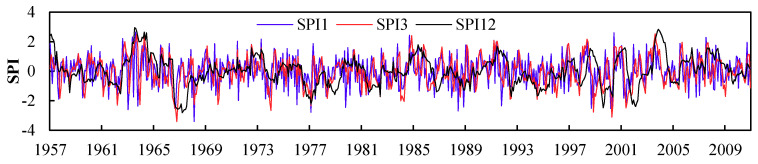
Variation of SPI series for different time scales in Northern Anhui province, 1957-2010.

Frequently, the SPI index can be applied to monitor the occurrence of drought, and drought will result in disaster when it occurs intensively and extensively in densely populated areas [31]. The overall variation trend of SPI series from one-month, three-month, and 12-month time scales for the Northern Anhui province through 1957 to 2010 was illustrated in Figure 4.

It can be revealed from Figure 4 that, (1) influenced by short-term precipitation events, the SPI1 series fluctuated significantly through 1957 to 2010, which was applied as the data basis to derive a different drought conceptual cloud, and then further explore the spatial evolution characteristics of drought in this study. (2) Concerning the consideration of precipitation in the previous three months, the variation of SPI3 series was relevant with agricultural drought [19,22]. The SPI12 series revealed the overall yearly variation trend of drought and waterlogging-related disasters. Therefore, the SPI3 and SPI12 series were applied to explore the temporal evolution characteristics of drought from seasonal and yearly scales in this study.

### 4.1. Temporal Variation Analysis of Drought

#### 4.1.1. Yearly Variation Characteristics Analysis

As indicated by SPI series in Figure 4, the drought evolution in the Northern Anhui province presented a slight decreasing trend from 1957 to 2010. If dividing drought intensity into four grades including light, moderate, severe, and extreme drought based on the indicator threshold of SPI12 proposed in meteorological drought classification criteria [5,6], the statistical result of drought duration of different grades on a monthly scale in the Northern Anhui province for each period was shown in Table 1.

Specifically, it can be indicated from Table 1 that, (1) the drought condition was the most serious from 1957 to 1970 in the Northern Anhui province in which a total of 49 months with SPI12 less than −0.5 and 12 months with an extreme drought level. The extreme drought duration was from August in 1966 to July in 1967. (2) The drought situation in the Northern Anhui province was relieved from 1971 to 1980, but the number of severe drought months increased from one from 1957 to 1970 to six from 1971 to 1980. (3) The drought evolution trend from 1981 to 1990 in the Northern Anhui province was approximately consistent with the period from 1971 to 1980. The drought situation was intensified from 1991 to 2000, but mainly presented with a light drought (18 months) and a moderate drought (22 months). (4) The drought situation in the Northern Anhui province was much relieved from 2001 to 2010. The total 49 months of drought occurred, but primarily presented a light drought level (19 months). (5) The statistical result of drought occurrence in Northern Anhui province from 1957 to 2010 was consistent with historical records [32], which verified the adaptability and reliability of applying the SPI drought index in drought recognition and distribution exploration to a large extent.

#### 4.1.2. Seasonal Variation Characteristics Analysis

Considering the impact of precipitation during the previous three months, the SPI3 series can be adopted to analyze the seasonal distribution features of drought. Therefore, the SPI3 characteristic value of spring, summer, autumn, and winter were obtained by the SPI3 of May, August, November, and next February separately, and then the SPI3 series from a seasonal scale for different cities in the Northern Anhui province can be constructed. In addition, the cloud theory was applied to further discuss the certainty and stability of seasonal distribution of drought in which the average *Ex* represented the overall intensity of drought. Entropy *En* revealed the certainty level of the determined drought intensity, and hyper-entropy *He* indicated the stability level about the variation trend of drought intensity. The higher of the entropy and hyper-entropy values, the more scattering distributed of cloud drops, and the higher uncertainty level of the cloud distribution pattern.

**Figure 5:**
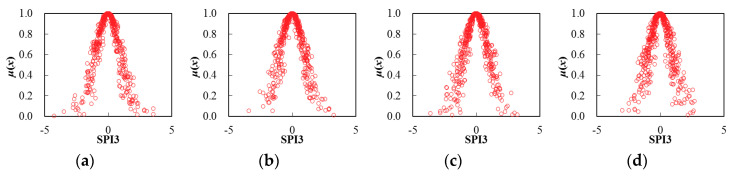
Cloud distribution of SPI3 in Huaibei, (**a**) spring, (**b**) summer, (**c**) autumn, and (**d**) winter.

**Figure 6:**
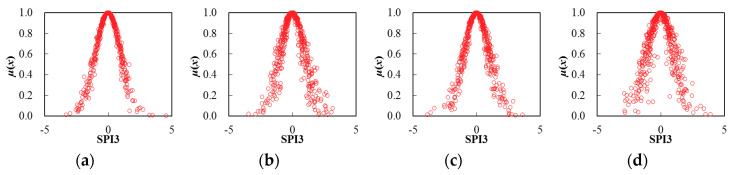
Cloud distribution of SPI3 in Bozhou, (**a**) spring, (**b**) summer, (**c**) autumn, and (**d**) winter.

**Figure 7:**
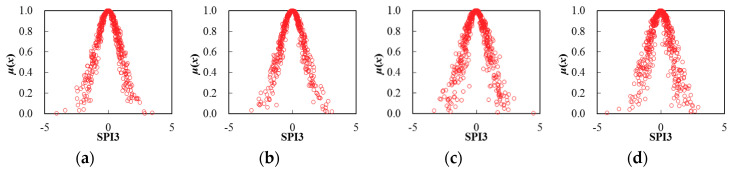
Cloud distribution of SPI3 in Suzhou, (**a**) spring, (**b**) summer; (**c**) autumn, and (**d**) winter.

**Figure 8:**
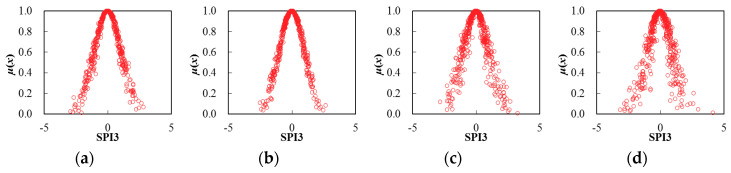
Cloud distribution of SPI3 in Bengbu, (**a**) spring, (**b**) summer, (**c**) autumn, and (**d**) winter.

**Figure 9:**
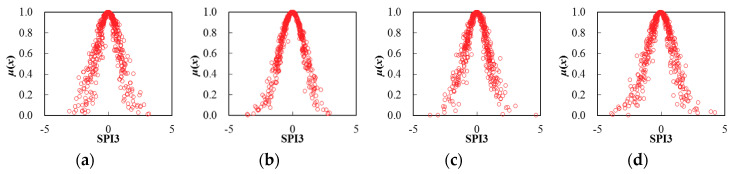
Cloud distribution of SPI3 in Fuyang, (**a**) spring, (**b**) summer, (**c**) autumn, and (**d**) winter.

**Figure 10:**
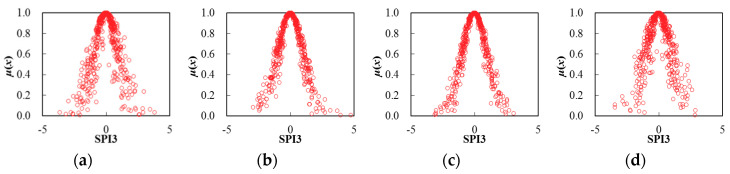
Cloud distribution of SPI3 in Huainan, (**a**) spring, (**b**) summer, (**c**) autumn, and (**d**) winter.

Thereby, based on the determination of cloud characteristic values for seasonal SPI3 series, the corresponding cloud distribution pattern can be obtained through the FNCA approach. Table 2 and Figure 5, Figure 6, Figure 7, Figure 8, Figure 9 and Figure 10 illustrated the seasonal cloud characteristic values and corresponding cloud distribution pattern, respectively, for different cities in the Northern Anhui province.

From Table 2 and Figure 5, Figure 6, Figure 7, Figure 8, Figure 9 and Figure 10, the temporal variation features of drought from a seasonal scale for different cities in the Northern Anhui province can be summarized as follows.

- Huaibei

The sorting result from high to low by the entropy values of seasonal SPI3 cloud distribution in Huaibei city was autumn, summer, spring, and winter. The sorting result by hyper-entropy values was winter, autumn, spring, and summer. Thus, it was evident that the fuzziness of drought distribution in autumn and summer was higher than that of spring and winter. In other words, the certainty of drought distribution in the winter was the highest in the Huaibei city. Moreover, the cloud layers indicating drought distribution in winter and autumn were much thicker than that in the spring and summer, and the stability of drought intensity and the variation trend in the summertime was the highest.

- Bozhou

The sorting result from high to low by the entropy values of seasonal SPI3 cloud distribution in Bozhou city was winter, spring, autumn, and summer, and the sorting result by hyper-entropy values was winter, summer, autumn, and spring. Thus, it was evident that the certainty level of drought distribution in the summer was the highest in Bozhou city. Furthermore, the stability of drought intensity and possible future variation trend in the spring was the highest.

- Suzhou

The sorting result from high to low by the entropy values of seasonal SPI3 cloud distribution in Suzhou city was the summer, autumn, spring, and winter, and the sorting result by hyper-entropy values was consistent with that of Huaibei city. Therefore, it was clear that the certainty level of drought distribution in the winter was the highest in Suzhou city. Moreover, the stability level of drought intensity and the future variation trend was consistent with that of the Huaibei city.

- Bengbu

The sorting result from high to low by the entropy values of seasonal SPI3 cloud distribution in Bengbu city was the spring, summer, autumn, and winter, which was exactly opposite with the sorting result by hyper-entropy values. Thus, it could be revealed that the uncertainty level of drought intensity and a possible evolution trend in the spring was the highest than during other seasons in Bengbu city.

- Fuyang

The sorting result from high to low by the entropy values of seasonal SPI3 cloud distribution in Fuyang city was winter, summer, spring, and autumn, and the sorting result by hyper-entropy values was spring, winter, autumn, and summer. Thus, it was evident that the uncertainty level of drought distribution in the winter was the highest, while the stability of a future variation trend of drought in the summer was the highest in Fuyang city.

- Huainan

The sorting result from high to low by the entropy values of seasonal SPI3 cloud distribution in Huainan city was winter, autumn, spring, and summer, and the sorting result by hyper-entropy values was winter, spring, summer, and autumn. Thus, it could be indicated that the fuzziness and randomness levels of drought distribution and future possible evolution trend in winter was the highest in Huainan city.

It could be revealed that the temporal variation characteristics of drought distribution in cities of Huaibei, Suzhou, and Bengbu were consistent with each other, with the highest certainty level of drought intensity distribution but lowest stability level of drought evolution trend in winter. This was exactly opposite with that of summer. The possible reasons causing the above distribution features of drought were that Huaibei, Suzhou, and Bengbu cities located in the eastern part of Anhui province, with little but stable annual precipitation influenced by a monsoon climate, which led to the stable and consistent seasonal distribution characteristics of drought.

### 4.2. Spatial Variation Characteristics Analysis of Drought

The cloud transformation algorithm (CTA) and conception zooming coupling approach was introduced to explore the spatial evolution characteristics of drought through re-fitting the distribution pattern of SPI3 series instead of Pearson-III distribution in this study. Concerning the similarity of the calculation process for different cities in the Northern Anhui province, the detailed determination procedures for cloud distribution characteristic values of drought through CTA and a conception zooming coupling approach in Huaibei city was illustrated as follows, which can be further applied in other cities of the Northern Anhui province.

- Determination of Conceptual Cloud Parameters

**Figure 11:**
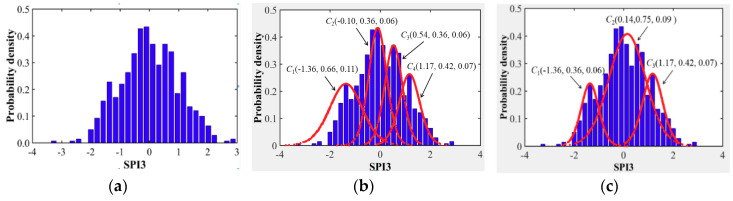
Determination process of cloud distribution of drought by CTA in Huaibei city, (**a**) probability density histogram, (**b**) initial conceptual cloud distribution, and (**c**) final cloud distribution of drought.

According to the procedures of CTA and conception zooming coupling approach indicated in Section 3.3, first, the calculated SPI3 series was applied to determine the probability density distribution histogram of drought, which was shown in Figure 11a. Then, supposing the threshold values ***δ*_1_** and ***δ*_2_** equaled to 0.1 and 0.01, respectively, which were used to control the occurrence frequency of remaining cloud drops, and the four initial conceptual cloud distributions of drought were obtained through the CTA method, as indicated in Figure 11b. Lastly, the ultimate three cloud distribution of drought for different drought intensity levels could be derived through a subtle adjustment of entropy values of initial drought cloud distribution and a merging process, which were illustrated in Figure 11c.

**Figure 12:**
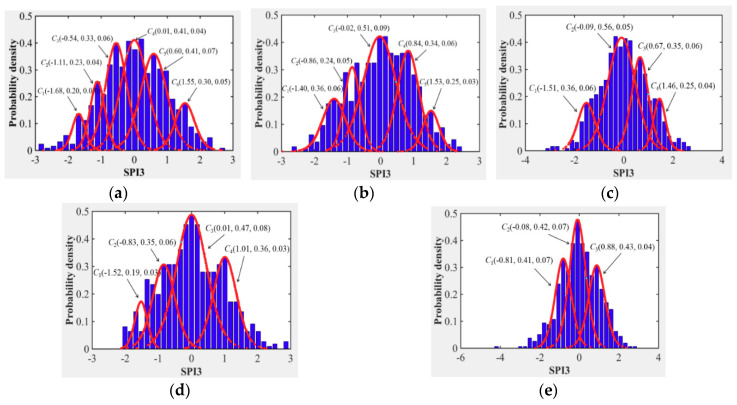
Conceptual cloud distribution of drought in Northern Anhui province, (**a**) Bozhou, (**b**) Suzhou, (**c**) Bengbu, (**d**) Fuyang, and (**e**) Huainan.

Similarly, the conceptual cloud distribution for different drought intensity in other cities of the Northern Anhui province could be derived by applying CTA and conception zooming coupling approach, and the cloud characteristic parameters and corresponding drought cloud distribution patterns were indicated in Table 3 and Figure 12.

- Evolution Trend Analysis of Concept Cloud for Drought

For the consistency with seasonal variation characteristics analysis of drought in Section 4.1.2, the average *Ex*, entropy *En*, and hyper-entropy *He* were also applied to present the overall intensity of drought and its derived certainty level and evolution stability level. Additionally, according to the division standard of the drought hazard based on the SPI index [22,33], the conceptual grade cloud distribution of drought for different cities in the Northern Anhui province under the same restrictions of cloud transformation principles and conceptual zooming threshold values, which could be obtained as indicated in Figure 11 and Figure 12. It can be evidently summarized from the two figures that:

(1) moderate grade cloud *C*_1_(−1.36, 0.36, 0.06) of drought occurred in Huaibei city, light, moderate, and severe grades cloud *C*_3_(−0.54, 0.33, 0.06), *C*_2_(−1.11, 0.23, 0.04), and *C*_1_(−1.68, 0.20, 0.03) occurred in Bozhou city, respectively, light and moderate grades cloud *C*_2_(−0.86, 0.24, 0.05) and *C*_1_(−1.40, 0.36, 0.06) of drought occurred in Suzhou city, respectively, severe grade cloud *C*_1_(−1.51, 0.36, 0.06) of drought occurred in Bengbu city, light and severe grades cloud *C*_2_(−0.83, 0.35, 0.06) and *C*_1_(−1.52, 0.19, 0.03) of drought occurred in Fuyang city, respectively, and light grade cloudC1(−0.81, 0.41, 0.07) of drought occurred in the Huainan city. 

(2) Among cities with the occurrence of a light grade cloud of drought (Bozhou, Suzhou, Fuyang, and Huainan), the certainty of determined drought intensity and stability of the corresponding drought evolution trend in Suzhou city was the highest, which was the opposite of Huainan city.

(3) Among cities with the occurrence of a moderate grade cloud of drought (Huaibei, Bozhou, and Suzhou), the certainty of determined drought intensity and stability of corresponding drought evolution trend in Buzhou city was the highest, which was opposite that in Huaibei city as well.

(4) Among cities with the occurrence of extreme grade cloud of drought (Bozhou Bengbu and Fuyang), the certainty of determined drought intensity and stability of the corresponding drought evolution trend in Fuyang city was the highest, which was also opposite that in Bengbu city.

Therefore, the drought hazard will be more intensified significantly along with the elevation of latitude in Northern Anhui province, not only for overall drought intensity at present but also for the evolution trend in the future. The overall drought hazard in Suzhou and Huaibei were the most serious followed by Bozhou, Bengbu, and Fuyang. Drought intensity in Huainan was the lightest.

## 5. Conclusions

For the influence of the monsoon climate, the northern Anhui province is one of the most typical regions with a large amount of drought-related disaster loss every year in mainland China. The purpose of this study is to explore the spatial-temporal distribution features of drought in the Northern Anhui province from both yearly and seasonal scales. The application results of the cloud transformation algorithm and conceptual zooming coupling model in the Northern Anhui province indicated that (1) the drought situation varying from 1957 to 1970 was the most serious during the study period, in which 12 months belong to an extreme drought level. (2) During the period from 1957 to 2010, the evolution trend of drought intensity varied with the highest certainty level but lowest stability level in the winter, but this was exactly opposite of that in the summer. (3) The overall drought intensity in Suzhou and Huaibei were the most serious from 1957 to 2010, which is followed by Bozhou, Bengbu, and Fuyang, and drought intensity in Huainan was the lightest. Thus, it can be deduced that the drought hazard would present a more severe trend with the elevation of latitude in the Northern Anhui province in the future. All in all, compared with previous work, the primary innovation of the manuscript is to essentially explore the spatio-temporal evolution characteristics of drought by transforming drought indicator series into multiple qualitative concepts of Gaussian cloud distribution based on the Cloud Transformation Algorithm (CTA) and Conception Zooming method. However, the reasonable computation of cloud characteristic parameters and the understanding of its transformation mechanism from a traditional interval threshold are crucial for systematic analysis, which is now inconclusive. Furthermore, drought is a complex natural phenomenon with randomness and uncertainties. How to further explore its natural evolution characteristics through comprehensive indicators integrating hydrological, meteorological, and underlying surface information is still a big challenge, which is exactly the foundation to realize the strategical planning research of drought risk management.

## Figures and Tables

**Table 1:** Statistical result of drought duration in the Northern Anhui province (unit: month).

Level	1957–1970	1971–1980	1981–1990	1991–2000	2001–2010
Light Drought	29	15	20	18	19
Moderate Drought	7	11	14	22	1
Severe Drought	1	6	1	8	7
Extreme Drought	12	2	0	0	0
Total	49	34	35	48	27

**Table 2:** Cloud characteristic values of SPI3 series for different seasons in the Northern Anhui province.

**Season**	**Huaibei**			**Bozhou**			**Suzhou**		
	***Ex***	***En***	***He***	***Ex***	***En***	***He***	***Ex***	***En***	***He***
Spring	−0.0045	0.9826	0.2278	−0.0003	1.0035	0.1104	0.0029	0.9947	0.1768
Summer	−0.0010	0.9846	0.2221	−0.0022	0.9854	0.2176	0.0004	1.0301	0.1635
Autumn	0.0020	1.0201	0.2372	0.0033	0.9976	0.1598	0.0027	1.0288	0.2393
Winter	−0.0198	0.9621	0.2742	−0.0066	1.0404	0.2738	−0.0027	0.9868	0.2484
**Season**	**Bengbu**			**Fuyang**			**Huainan**		
	***Ex***	***En***	***He***	***Ex***	***En***	***He***	***Ex***	***En***	***He***
Spring	0.0003	1.0484	0.1279	0.0053	0.9700	0.2260	0.0026	0.9689	0.2852
Summer	0.0017	1.0041	0.1061	0.0007	1.0112	0.1385	0.0014	0.9944	0.1754
Autumn	0.0017	0.9854	0.2208	0.0073	0.9657	0.2108	−0.0001	0.9956	0.1674
Winter	−0.0170	0.9838	0.2317	−0.0227	1.0195	0.2169	−0.0177	1.0241	0.3200

**Table 3:** Cloud characteristic parameters of drought in the Northern Anhui province.

**Value**	**Huaibei**			**Bozhou**						**Suzhou**				
	***C*_1_**	***C*_2_**	***C*_3_**	***C*_1_**	***C*_2_**	***C*_3_**	***C*_4_**	***C*_5_**	***C*_6_**	***C*_1_**	***C*_2_**	***C*_3_**	***C*_4_**	***C*_5_**
*Ex*	−1.36	0.14	1.17	−1.68	−1.11	−0.54	0.01	0.60	1.55	−1.40	−0.86	−0.02	0.84	1.53
*En*	0.36	0.75	0.42	0.20	0.23	0.33	0.41	0.41	0.30	0.36	0.24	0.51	0.34	0.25
*He*	0.06	0.09	0.07	0.03	0.04	0.06	0.04	0.07	0.05	0.06	0.05	0.09	0.06	0.03
**Value**	**Bengbu**				**Fuyang**				**Huainan**					
	***C*_1_**	***C*_2_**	***C*_3_**	***C*_4_**	***C*_1_**	***C*_2_**	***C*_3_**	***C*_4_**	***C*_1_**	***C*_2_**	***C*_3_**			
*Ex*	−1.51	−0.09	0.67	1.46	−1.52	−0.83	0.01	1.01	−0.81	−0.08	0.88			
*En*	0.36	0.56	0.35	0.25	0.19	0.35	0.47	0.36	0.41	0.42	0.43			
*He*	0.06	0.05	0.06	0.04	0.03	0.06	0.08	0.03	0.07	0.07	0.04			
